# Estimation of the Age and Amount of Brown Rice Plant Hoppers Based on Bionic Electronic Nose Use

**DOI:** 10.3390/s141018114

**Published:** 2014-09-29

**Authors:** Sai Xu, Zhiyan Zhou, Huazhong Lu, Xiwen Luo, Yubin Lan, Yang Zhang, Yanfang Li

**Affiliations:** 1 Key Laboratory of Key Technology on Agricultural Machine and Equipment, South China Agricultural University, Guangzhou 510642, China; E-Mails: xusai1991@sina.cn (S.X.); huazlu@scau.edu.cn (H.L.); xwluo@scau.edu.cn (X.L.); ylan@tamu.edu (Y.L.); 2 College of Engineering, South China Agricultural University, Guangzhou 510642, China; 3 Department of Biological and Agricultural Engineering, Texas A&M University, College Station, TX 77843, USA; 4 Plant Protection Research Institute, Guangdong Academy of Agricultural Sciences, Guangzhou 510640, China; E-Mails: zhangy@gdppri.com (Y.Z.); liyf458@tom.com (Y.L.); 5 Guangdong Provincial Key Laboratory of High Technology for Plant Protection, Guangzhou 510640, China

**Keywords:** bionic electronic nose, bionic olfaction, brown rice plant hopper, age, amount, volatile, classification

## Abstract

The brown rice plant hopper (BRPH), *Nilaparvata lugens* (Stal), is one of the most important insect pests affecting rice and causes serious damage to the yield and quality of rice plants in Asia. This study used bionic electronic nose technology to sample BRPH volatiles, which vary in age and amount. Principal component analysis (PCA), linear discrimination analysis (LDA), probabilistic neural network (PNN), BP neural network (BPNN) and loading analysis (Loadings) techniques were used to analyze the sampling data. The results indicate that the PCA and LDA classification ability is poor, but the LDA classification displays superior performance relative to PCA. When a PNN was used to evaluate the BRPH age and amount, the classification rates of the training set were 100% and 96.67%, respectively, and the classification rates of the test set were 90.67% and 64.67%, respectively. When BPNN was used for the evaluation of the BRPH age and amount, the classification accuracies of the training set were 100% and 48.93%, respectively, and the classification accuracies of the test set were 96.67% and 47.33%, respectively. Loadings for BRPH volatiles indicate that the main elements of BRPHs' volatiles are sulfur-containing organics, aromatics, sulfur- and chlorine-containing organics and nitrogen oxides, which provide a reference for sensors chosen when exploited in specialized BRPH identification devices. This research proves the feasibility and broad application prospects of bionic electronic noses for BRPH recognition.

## 1. Introduction

As one of the main food crops in China, rice plays an important role in China's food safety guarantee system and agricultural production. In addition, China's rice planting area accounts for approximately 19% of the world's total [1]. However, biological disasters are one of the main factors affecting stable and high rice yields. A total of four to five million tons of rice grain are damaged by biological disasters each year [2]. Brown rice plant hopper (BRPH), a migratory pest, is one of the main pests that affect stable and high yields of rice. Under appropriate conditions, BRPH disasters can occur after a month of breeding, and the insects can reproduce an entire generation within that time, which causes huge grain waste and economic loss [3]. Thus, methods to prevent and ameliorate BRPH damage have become a hot research issue in recent years [4,5]. Only the acquisition of accurate pest information can facilitate prevention and successful treatment. Using such information, we can forecast the pest damage according to pest information and remediate the damage in the earliest stages. In addition, the quantity of pesticide spraying can be decided by the use of pest information, which facilitates effective pesticide use. Currently, there are several methods to determine BRPH information, including the manual work detection method [6], the acoustical signal detection method [7,8], the radar observation method [9], the image recognition method [10] and the near-infrared spectroscopy method [11]. The manual work detection method mainly depends on human sense organs to detect pest information subjectively, which has low detection efficiency, high detection cost and huge labor intensity, and is often influenced by sampling. Machine detection methods, such as the acoustical signal detection method, the radar observation method, the image recognition method and the near-infrared spectroscopy method, reduce labor intensity to some extent, but as the field environment is complex, pest detection can easily be influenced by the weather, light intensity, pest mobility and masking, resulting in the methods being unable to meet the needs of practical production. Thus, it is important to explore a new BRPH detection method.

As a bionic detection method, the bionic electronic nose can analyze, identify and detect complex smells and most volatile gases. Compared with common chemical analysis instruments that can only provide quantitative results from one or certain types of elements of a sample, the output data of a bionic electronic nose can obtain the whole information of the volatile gases of the sample, namely “fingerprint” data [12]. With the characteristics of rapid detection, no solvent requirements, insusceptibility to environmental disturbances, such as light intensity and pest mobility or masking, the ease of carrying and more objectivity than the human sense of smell, the bionic electronic nose is widely used in environmental monitoring [13,14], biological bacteria detection [15,16], food quality detection [17,18] and medical treatment and health [19,20]. The bionic electronic nose has also been used in BRPH detection. In 2005, Ye and Hu used an electronic nose (PEN2) to detect the volatile blends of paddy rice and to classify volatile odors of BRPHs. The experiment indicated that the best opportunity for detecting pest information using an electronic nose is from 15 to 36 h after pest damage. These findings demonstrated that electronic noses are an efficient method for obtaining rice pest information [21]. In 2006, Hu applied an electronic nose to detect BRPH information based on the obtained sensor array data. The feature parameters from each sensor curve, including maximum, max differential value, mean value and stable value, were extracted and then used for pattern recognition input. Then, principal component analysis (PCA) was adopted to analyze the test sample. The experiments revealed that the electronic nose was able to detect whether rice was attacked by insect pests, the extent of rice damage and the number of pests on each stem of paddy rice [22]. Zhou and Wang used an electronic nose (PEN2) to discriminate between the volatile profiles emitted by uninjured rice plants and those emitted by rice plants exposed to different numbers of BRPH adults. The results showed that the electronic nose can separate different rice plants effectively, which has broad applying prospects for insect monitoring [23]. In 2011, Zhou and Wang used an electronic nose (PEN2) to analyze rice plants that were subjected to different types of treatments causing damage, including damage caused by the rice striped stem borer, damage caused by the BRPH and mechanical damage, and the results were compared to that of undamaged control plants. The results indicated that the e-nose can successfully discriminate between rice plants with different types of damage [24], but BRPH information detection based on pest body volatile odors using an electronic nose has not been reported until now.

In this article, we sample different numbers and ages of BRPHs using an electronic nose (PEN3). In addition, principal component analysis (PCA), linear discrimination analysis (LDA), probabilistic neural network (PNN), BP neural network (BPNN) and loading analysis (Loadings) techniques were used to analyze the sample data with the aim of evaluating the feasibility of detecting the age and number of BRPHs by the electronic nose and providing a further scientific reference for brown rice plant hopper forecasting.

## 2. Materials and Methods

### 2.1. Experiment Materials and Grouping

All of the experimental BRPHs were provided by the Institute of Plant Protection of the Academy of Agricultural Sciences in Guangzhou, Guangdong province, China. The grouping of rice plant hopper damage was based on the Chinese national standard (Table 1) divided by 100. The experimental BRPHs were divided into 3 age stages, namely younger than 3rd-instar nymphs (U3IN), older than 3rd-instar nymphs (O3IN) and adults. There were five groups for each age stage: 5P (five pests in each sample), 10P (10 pests in each sample), 20P (20 pests in each sample), 30P (30 pests in each sample) and 50P (50 pests in each sample). Ten samples were prepared for each group. Before sampling, all of the samples were sealed in 11-mL gas collected bottles for 1 h (the environment temperature is 26°C, environment humidity is 52%). In addition, five empty gas collecting bottles were used for comparison. Thus, there were a total of 155 samples (3 ages × 5 groups ×10 samples per group + 5 empty gas collecting bottle samples = 155 samples). Before the gas collecting bottles were used, ultrasonic cleaning instruments were used to clean them and to dry naturally in an indoor environment without an obvious smell.

### 2.2. Electronic Nose Set-Up

**Figure 1.**
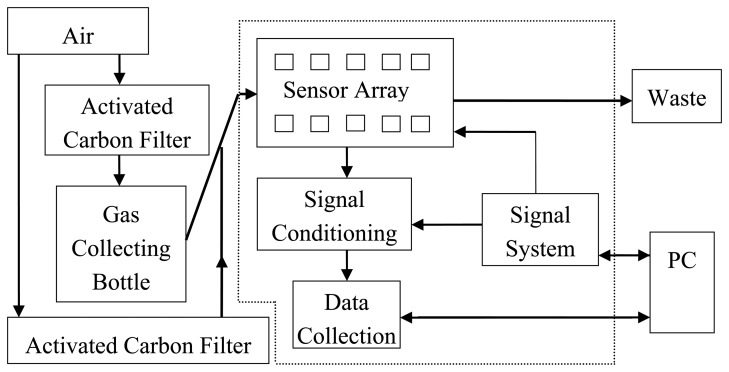
The structure of the electronic nose (PEN3).

An electronic nose (PEN3, Airsense Analytics GmbH, Schwerin, Germany) was used in this experiment for sampling. Its system structure is shown in Figure 1. A sensor array, a signal preprocessing unit and a pattern recognition algorithm are the main components of the electronic nose. The sensor array contains 10 different sensors, and each sensor is sensitive to different volatile types. The testing principle of the electronic nose is that a transient response (a series of physical and chemical changes) will occur when the active material of the sensors contacts the volatiles. After magnifying the response, it was translated from the voltage signal to a figure signal via the signal preprocessing unit, which is recorded by a PC and transmitted to a signal processing system for analysis. Finally, the signal processing outcomes are evaluated by a pattern recognition subsystem to obtain the results [25].

**Figure 2.**
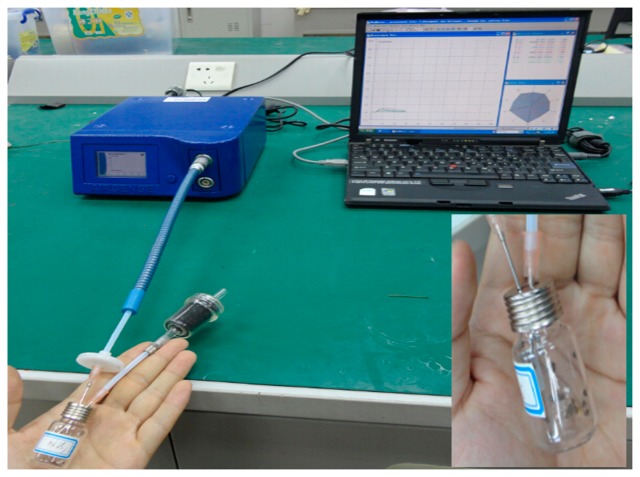
Used the electronic nose for brown rice plant hopper (BRPH) sampling.

The electronic nose for BRPH sampling is shown in Figure 2. In order to ensure that the sensors reached their working temperature, the electronic nose was preheated for 10 min before the measurement was performed. Before sampling, the sensor array of the electronic nose was flushed by zero gas (the field air, which is filtered by an activated carbon filter). The working parameter settings are shown in Table 2.

### 2.3. Neural Networks

Linear classification methods, such as PCA and LDA, typically cannot achieve ideal classification results [26]. Thus, to further research the ability of electronic noses to classify the BRPH, we should use nonlinear methods (neural network methods are used in this paper) for analysis. Neural networks can be classified into different types based on their structure. Each type has both advantages and disadvantages. This paper uses PNN and BPNN for further analysis.

#### 2.3.1. Probabilistic Neural Network

The probabilistic neural network (PNN) was first introduced by Specht in 1989 [27]. The PNN is a Bayes–Parzen classifier. The foundation of the approach was established decades ago; however, the method was not in widespread use, because of the lack of sufficient computation power until recently [28]. A PNN is composed of an input layer, a pattern layer, a summation layer and an output layer [29]. The input layer passes the feature vectors of training samples to the network through a linear transfer function, and the number of neurons in the input layer is equal to the dimension of the training samples. The pattern layer calculates the matching relation between input feature vectors and each pattern in the training, and the number of neurons in the pattern layer is equal to the sum of each category of training samples; the output of each pattern unit is a non-linear operator. The summation layer simply adds the outputs which belong to their own class of pattern layer units. The output of the summation layer unit is proportional to the probability density estimation of each class. Through normalization processing in the output layer, the probability estimation of each class can be obtained. The fourth layer is the output layer, which is composed of a simple threshold discriminator, whose role is to choose the neuron with the maximum posterior probability density in each model as the output of the whole system. The neuron with the biggest posterior probability density outputs 1, which corresponding to this class, is the pattern category for identifying the sample; and other neurons' output is 0 [30].

The function toolbox of MATLAB [31] was used for the PNN analysis in this research. In addition, the value of the spread is the diffusion rate of the PNN model that can be optimized for maximum classification accuracy, and its default is 0.1.

#### 2.3.2. BP Neural Network

The BP neural network (BPNN) is one of the most commonly used neural networks, which includes an input layer, a hidden layer and an output layer. In the process of training BPNN for analysis, the weights and threshold values of each layer are revised constantly. The BPNN adjusts the weights and threshold values repeatedly based on the difference between the expected outputs and actual outputs. That is to say, the BPNN is a neural network that spreads information in the forward direction and returns the difference in the reverse direction. This training lasts until the difference between the expected outputs and actual outputs is limited to a preliminary range or the scheduled training times are achieved [32].

### 2.4. Feature Extraction

#### 2.4.1. Feature Extraction for PCA, LDA and Loadings

**Figure 3.**
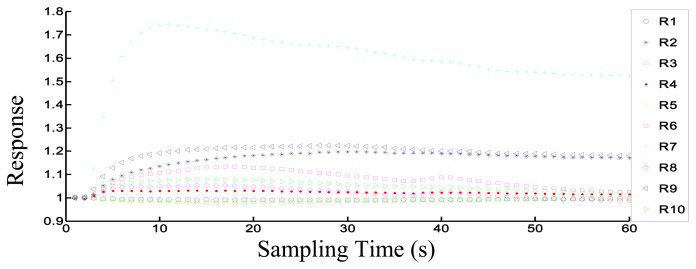
The response of the electronic nose to 30 BRPH adults (where R1–R10 represent the 10 sensors, respectively).

Figure 3 shows the response of the electronic nose to the 30P sample. The *i*-th (from 1 to 10) sensor's response data *R_i_* is the ratio of the resistance value *G* (when sensors contact sample volatiles) and the resistance value *G_0_* (when sensors contact zero gas). The value of each sensor is 1 in the initial state (0 s). In this figure, after the volatiles contact each sensor, the sensor signal changes greatly. After 50 s, the response curve of each sensor approaches the steady state. We chose each sensor's steady-state response value for analysis. Thus, the response value in the 52nd second was chosen for the PCA, LDA and Loading analysis in this experiment.

#### 2.4.2. Feature Extraction for PNN and BPNN

Feature extraction should contain as much sample information as possible. Different sensors have different response values and response rates. Thus, for this experiment, we chose the average differential value *D_ave_* and average value *S_ave_* of the whole response value of each sensor for PNN and BPNN. The computational formulas are as follows:

(1) $$D_{\text{ave}}=\frac{1}{n-1}\sum_{z=1}^{n-1}\frac{x_{z+1}-x_{z}}{\Delta t}$$

where n is the amount of the test point (*n*=60), *x_z_* is the *z*-th response value of a sample and *Δt* is the time difference of adjacent test points (*Δt* = 1s).

(2) $$S_{\text{ave}}=\frac{1}{60}\sum_{i=1}^{60}x_{i}$$

where *x_i_* is the *i*-th response value of a sample.

### 2.5. Data Processing

For BRPH age classification, the measured electronic nose data were classified into three groups, namely, U3IN, O3IN and adults. Then, PCA, LDA, PNN and BPNN were used for analysis and classification with the aim of judging the classification effect of estimating the BRPH age using the electronic nose.

For BRPH amount classification, the measured electronic nose data were classified into six groups, namely 5P, 10P, 20P, 30P and 50P. Then, PCA, LDA, PNN and BPNN were used for analysis and classification, with the aim of judging the classification effect of estimating the amount of BRPH using the electronic nose.

For analyzing volatiles of BRPH, Loadings were used for analyzing the sensors, which are mainly sensitive to the volatiles of BRPH and indicate the main components of the volatiles of BRPH.

K-fold cross-validation [33] was used for PNN and BPNN analysis. In K-fold cross-validation, the original sample is randomly partitioned into K subsamples. Of the K subsamples, a single subsample is retained as the validation data for testing the model, and the remaining K−1 subsamples are used as training data. The cross-validation process is then repeated K times (the folds), with each of the K subsamples being used exactly once as the validation data. The K results from the folds then can be averaged (or otherwise combined) to produce a single estimation. The advantage of this method over repeated random sub-sampling is that all observations are used for both training and validation, and each observation is used for validation exactly once.

## 3. Results and Discussion

### 3.1. PCA and LDA Method

#### 3.1.1. PCA and LDA for BRPH Age Estimation

**Figure 4.**
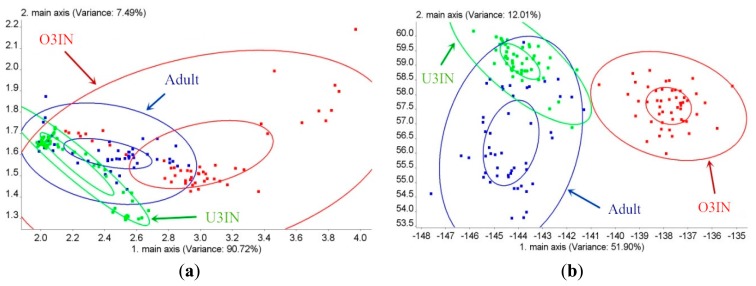
Age classification of BRPH: (**a**) PCA for age classification of BRPH;(**b**) LDA for age classification of BRPH.

The age classification results for BRPH using PCA are shown in Figure 4a. The contribution of the first principal component (PC1) is 90.72%, and the contribution of PC2 is 7.49%. Thus, the cumulative contribution is 98.21%. In this figure, all of the three ages overlap each other. Thus, the BRPH age cannot be classified via PCA. The age classification results for BRPH using LDA are shown in Figure 4b. The contribution of the first linear discrimination (LD1) is 51.9%, and the contribution of LD2 is 12.01%; the cumulative contribution is 63.91%. This figure shows that the O3IN group can be classified with the other two ages via LDA. However, the U3IN group overlaps with the adult group. Both the U3IN and adult groups cannot be classified. Thus, the BRPH cannot be estimated using PCA and LDA, and the age classification effect of LDA is superior to that of PCA.

#### 3.1.2. PCA and LDA for BRPH Amount Estimation

**Figure 5.**
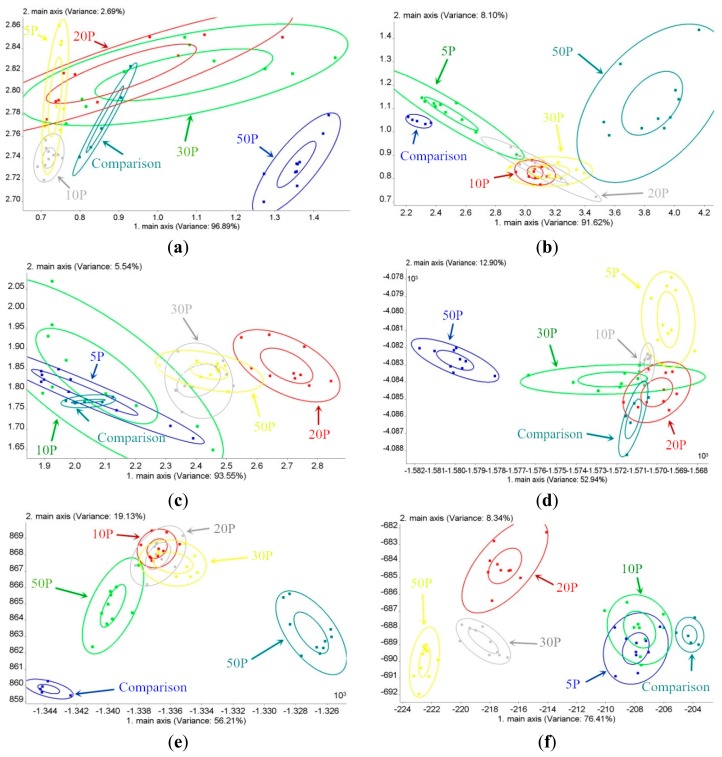
BRPH amount classification for different ages: (**a**) PCA for the amount classification of the younger than 3rd-instar nymphs (U3IN) group; (**b**) PCA for the amount classification of the older than 3rd-instar nymphs (O3IN) group; (**c**) PCA for the amount classification of the adult group; (**d**) LDA for the amount classification of the U3IN group;(**e**) LDA for the amount classification of the O3IN group; (**f**) LDA for the amount classification of the adult group.

The contributions of PC1, PC2, LD1 and LD2 for different groups with varying amounts of BRPHs of different ages are shown in Table 3. The maximum cumulative percentage of PC1 and PC2 is observed with the O3IN group (99.72%), and the second largest one is the U3IN group (99.58%). The minimum one is the adult group (99.09%). The classification results for the U3IN, O3IN and adult BRPH groups using PCA are shown in Figure 5a–c, respectively. The 50P group of U3IN can be classified with other groups of U3IN via PCA. The comparable group of O3IN can be classified with other groups of O3IN via PCA, but the BRPH amount groups for the adult BRPH cannot be classified. The classification effect for the O3IN and U3IN groups is superior to that of for the adult group, which is in direct proportion to the cumulative PCs contribution.

According to Table 3, the maximum cumulative percentage of LD1 and LD2 is observed with the adult group (84.75%). The second largest cumulative percentage of LD1 and LD2 is observed with the O3IN group (75.34%), and the minimum cumulative percentage of LD1 and LD2 is observed with the U3IN group (65.84%). The classification results for the U3IN, O3IN and adult BRPH groups using LDA are shown in Figure 5d–f, respectively. The results prove that the 50P group of U3IN can be classified with other groups of U3IN via LDA. In addition, both the comparable group and the 50P O3IN group can be classified with other groups of O3IN via LDA. Additionally, all of the amounts of the groups of adult BRPH can be classified, except for the 5P and 10P groups. The LDA classification effect is best for the adult group, with O3IN in the second place, and U3IN being the worst, which is in direct proportion to the cumulative contribution of the LDs. In addition, the LDA classification performance is superior to that of PCA.

### 3.2. Probabilistic Neural Network

#### 3.2.1. PNN for BRPH Age Estimation

To further research the classification ability of the electronic nose concerning BRPH age, PNN was used to classify different BRPH ages. Five-fold cross-validation was used for the training set and test set selection. We divided all samples into five equal groups (each group has 30 samples) at random, four groups as the training set and the remaining group as the test set. There were 120 training samples and 30 test samples in total. There were 20 neurons (equal to the number of feature value of each sample) in the input layer and three neurons (equal to the number of classes) in the output layer. To optimize the PNN network model, we chose a spread in the optimal range of [1×10^−3^, 2×10^−3^, 3×10^−3^, 4×10^−3^, 5×10^−3^, 6×10^−3^, 7×10^−3^, 8×10^−3^, 9×10^−3^, 1 ×10^−2^]. This research chose the best model where both the classification accuracy of the training set and the test set were the highest at the same time. After five-times analysis, the classification results are shown in Table 4. The training set's average classification accuracy is 100%, and the test set's average classification accuracy is 90.67%. Thus, PNN can provide good classification for BRPH age estimation.

#### 3.2.2. PNN for BRPH Amount Estimation

For the BRPH amount classification via PNN, six-fold cross-validation was used for the training set and test set selection. We divided all samples into six equal groups (each group has 25 samples) at random, five groups as the training set and the remaining group as the test set. There were 125 training samples and 25 test samples in total. There were 20 neurons in the input layer and five neurons in the output layer. To optimize the PNN network model, we chose a spread in the optimal range of [1×10^−3^, 2×10^−3^, 3×10^−3^, 4×10^−3^, 5×10^−3^, 6×10^−3^, 7×10^−3^, 8×10^−3^, 9×10^−3^, 1×10^−2^]. This study used the best model, which was defined as the model where both the classification accuracy of the training set and the test set were simultaneously the highest. After six-times analysis, the classification results are shown in Table 4. The training set's average classification accuracy was 96.67%, and the test set's average classification accuracy was 64.67%. Thus, the classification performance of PNN for the BRPH amount estimation is poor.

### 3.3. BP Neural Network

#### 3.3.1. BPNN for BRPH Age Estimation

For BRPH age classification via BPNN, we used the same training set and test set as the PNN analysis for BRPH age classification in this research. Thus, there were 120 training samples and 30 test samples in total. There were 20 neurons in the input layer and three neurons in the output layer. The transfer function of BPNN is y=tansig(x), and y and x are the output value and the input value, respectively. The BPNN analysis set the expected output of the U3IN, O3IN and adult groups to (0, 0), (0, 1) and (1, 1), respectively. After repeated debugging, the classification results and corresponding parameter setting (the number of nodes in the hidden layer, the learning rate, the dynamic factor and maximum number of training times) are shown in Table 5. After repeating five times, the classification function was trained using the training set, in which the average back classification ratio was 100%. Then, the classification function was tested using the test set, in which the average back classification ratio was 96.67%. These results indicate that BPNN can achieve good classification in the age estimation of BRPHs.

#### 3.3.2. BPNN for BRPH Amount Estimation

For BRPH amount classification via BPNN, we used the same training set and test set as the PNN analysis for the BRPH amount classification in this research. Thus, there were 120 training samples and 30 test samples in total. There were 20 neurons in the input layer and five neurons in the output layer. The transfer function of BPNN is y=tansig(x), and y and x are the output value and the input value, respectively. The BPNN analysis set the expected output of the 5P, 10P, 20P, 30P and 50P groups to (1, 0, 0, 0, 0), (0, 1, 0, 0, 0), (0, 0, 1, 0, 0), (0, 0, 0, 1, 0) and (0, 0, 0, 0, 1), respectively. After repeated debugging, the classification results and corresponding parameter setting (the number of nodes in the hidden layer, the learning rate, the dynamic factor and the maximum number of training times) are shown in Table 5. After repeating six times, the classification function was trained using the training set, which had a back average classification ratio of 48.93%. Then, the classification function of the test set was tested, and its back average classification ratio was 47.33%. Thus, the classification performance for the BRPH amount estimation via the BPNN was poor.

### 3.4. Loading Analysis Method

**Figure 6.**
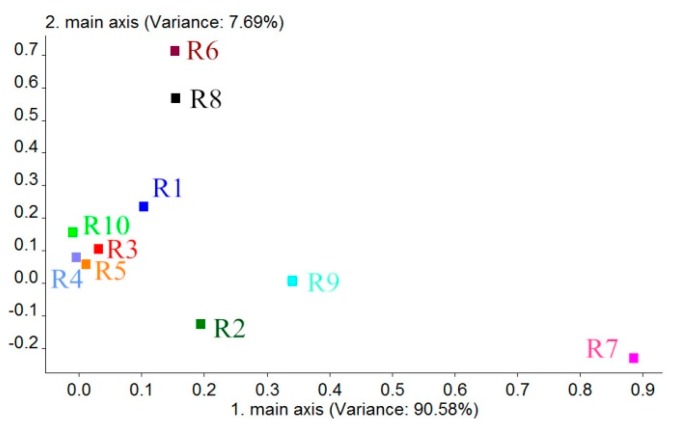
Loadings for BRPH volatiles.

A loading is the correlation coefficient of a PC and the corresponding original variables. Loadings can reflect the intimacy levels of each PC or variable and be used to judge the contribution and correlation of each sensor to PC1 and PC2 [34,35]. According to this, we can get the coordinate of each sensor in a Loadings plot by the first correlation coefficient (the first loading factor) and the second correlation coefficient (the second loading factor), which can evaluate which sensors are useful or not useful for classification and analyzing the classification ability of each sensor. Generally, when the coordinate value of a sensor is farther from the original point (0, 0), the sensor's contribution for sample classification is larger. The results for the Loadings are shown in Figure 6. According to this figure, the contribution rate of PC1 was 90.58%. The contribution rate of PC2 is 7.69%, which is much lower than that of PC1. Thus, on the basis of the loading distribution of each sensor in PC1, we can obtain results that indicate that the sensor's contributions to BRPH classification are R7, R9 and R2, respectively. The contribution of other sensors is smaller. Based on the sensitive materials of each sensor, which are shown in Table 6, we can infer that the main components of the BRPHs' volatiles are sulfur-containing organics (R7), aromatics, sulfur- and chlorine-containing organics (R9) and nitrogen oxides (R2). The results also provide a reference for the sensors chosen. We can choose and exploit the sensors that are sensitive to the above main BRPH volatile components in specialized BRPH identification devices.

### 3.5. Discussion

The purpose of this study was to research the feasibility of using an electronic nose to estimate the age and amount of BRPHs. The research results previously discussed indicate that an electronic nose can effectively estimate the age of BRPHs. However, the performance of the electronic nose when estimating the amount of BRPHs is poor.

BRPHs of different ages have different volumes of honeydew, and the volume of honeydew produced by the BRPHs with different physiological features (macropterous, brachypterous, male and female) is also different [36]. This may be the reason that the age classification performance is good. However, when using the electronic nose to classify the BRPH amounts, there were many interference factors that may have influenced the classification results, including age, being macropterous, being brachypterous, being male or being female (their volumes of honeydew differ). Thus, the amount classification performance was poor. As the age classification effect performance was good despite the interference of other factors, we can infer that age is the main factor that can affect the electronic nose's classification performance for BRPH.

The classification performances for both PCA and LDA were poor, but the LDA classification performance was better than that of PCA. The classification performance of PCA is based on the sample's distribution in two-dimensional space, which may be composed of PC1 and PC2, but LDA focuses more on the distribution and the distance within each group. It can collect data information from whole sensors and obtain each group via a particular vectorization transformation, which results in the samples within a group being condensed and distant samples in different groups. Thus, LDA could achieve a better classification performance than PCA. In addition, when the calculative contribution is higher, more original information is contained by PCs or LDs. Thus, the classification effect is in direct proportion to the cumulative contribution of the PCs or LDs

PNN and BPNN are useful for BRPH age classification, but the BRPH amount classification performance via either a PNN or BPNN was poor. Both the PNN and BPNN are better than LDA and PCA for BRHP classification. The neural network (NN) method, which includes the PNN and BPNN methods, is a nonlinear classification method that can achieve arbitrary nonlinear mapping from input to output. NN displays good performance when applied to functional mapping, functional approximation and self-adaption. Thus, the PNN and BPNN are better than PCA and LDA for nonlinear problems, such as BRPH classification.

The results of this study prove the tentative feasibility of using electronic noses for the age classification of BRPH, but the BRPH amount classification performance was poor. However, there are still a number of potential problems associated with the application of electric noses for the classification of BRPH age and amount classification. First, the electronic nose is sensitive to changes in the humidity and temperature, which can greatly affect the outputs of electronic noses. Although the humidity and temperature changes are small in the actual test, follow-up additional research focusing on how to reduce the influence of humidity and temperature change in the experiment environment is very significant for further improvement of classification accuracy. Secondly, the volatile change of samples caused by additional factors, such as being macropterous, being brachypterous or sex, should be taken into consideration in the next study. Third, there is also a need to reduce the number of sensors in the sensor array to reduce the cost of the electronic noses. In this article, the BRPH loading results aid us in choosing sensors that are sensitive to sulfur-containing organics, aromatics, sulfur- and chlorine-containing organics and nitrogen oxides for BRPH classification. Moreover, software improvements may resolve some of the problems.

## 4. Conclusions

This research used a PEN3 electronic nose to estimate the age and number of BRPHs. This research also used PCA, LDA, PNN, BPNN and Loadings for analysis. The results of this research prove the feasibility of the use of bionic electronic noses for BRPH recognition and that electronic noses have broad prospects for application in BRPH information estimation. The experimental results are as follows.

(1) The classification performances of PCA and LDA are proportional to the contributions of the sensors. The classification performances for both PCA and LDA are poor.
(2) The classification performances of the tested NNs (PNN and BPNN) are better than those of LDA and PCA, and the classification performance of LDA is better than that of PCA for BRPH classification.
(3) PNN can classify the age of BRPHs accurately, and the classification accuracy for the training and test sets are 100% and 90.67%, respectively. The classification performance is poor when a PNN is used for BRPH amount classification, and the classification accuracy of the training and test sets are 96.67% and 64.67%, respectively.
(4) BPNN can also accurately classify the age of BRPHs. After training the BPNN model, the classification accuracy of the training and test sets are 100% and 96.67%, respectively. The classification performance for the BRPH amount classification using a BPNN is poor, and the classification accuracy of the training and test sets are 48.93% and 47.33%, respectively.
(5) Loading results indicate that the main elements of the BRPHs' volatiles are sulfur-containing organics (R7), aromatics, sulfur- and chlorine-containing organics (R9) and nitrogen oxides (R2). The results also provide a reference of the sensors to choose. We can choose the sensors that are sensitive to the aforementioned molecules for exploitation in a specialized BRPH identification device.
(6) We can infer that age is the main factor that can affect BRPH classification when using an electronic nose.

## Figures and Tables

**Table 1.** Rice plant hopper infestation rating.

**Damage Degree**	**Undamaged**	**Light**	**Middle**	**Serious**
Pest number	0	<1000	1000–3000	>3000

**Table 2.** The working parameter settings of the electronic nose.

**Working Parameters**	**Sampling Interval**	**Flush Time**	**Zero Point Trim Time**	**Measurement Time**	**Pre-Sampling Time**	**Injection Flow**
Values	1 s	60 s	10 s	60 s	5 s	300 mL/min

**Table 3.** The contribution of PCs or LDs for different amounts of BRPHs of different ages.

**Age**	**PC or LD**	**PCA**		**LDA**	
	
		**Contribution**	**Cumulative Contribution**	**Contribution**	**Cumulative Contribution**
U3IN	1	96.89%	99.58%	52.94%	65.84%
	2	2.69%		12.9%	

O3IN	1	91.62%	99.72%	56.21%	75.34%
	2	8.10%		19.13%	

Adult	1	93.55%	99.09%	76.41%	84.75%
	2	5.54%		8.34%	

Notes: U3IN, under the 3rd-instar nymphs; O3IN, over the 3rd-instar nymphs. There were a total of 155 samples for the cross-test, which includes 50 samples for each age and five samples for comparison.

**Table 4.** PNN for BRPH age and amount classification based on K-fold cross-validation.

	**PNN for BRPH Age Classification**			**PNN for BRPH Amount Classification**		

**Repeat Times**	**Classification Accuracy**		**Spread**	**Classification Accuracy**		**Spread**
	
	**Training Set**	**Test Set**		**Training Set**	**Test Set**	
1	100%	96.67%	0.004	100%	60%	0.003
2	100%	93.33%	0.004	96%	80%	0.004
3	100%	86.67%	0.002	96%	64%	0.002
4	100%	90%	0.003	96%	64%	0.008
5	100%	86.67%	0.008	96%	60%	0.002
6				96%	60%	0.001

Average accuracy	100%	90.67%		96.67%	64.67%	

**Table 5.** BPNN for BRPH age and amount classification based on K-fold cross-validation.

	**Repeat Times**	**Nodes**	**LR**	**DF**	**MTT**	**Classification Accuracy**	

						**Training Set**	**Test Set**
BPNN for BRPH age classification	1	18	0.035	0.05	20,000	100%	100%
	2	18	0.035	0.05	20,000	100%	96.67%
	3	18	0.034	0.05	20,000	100%	96.67%
	4	19	0.034	0.05	20,000	100%	100%
	5	19	0.036	0.05	20,000	100%	90%

Average accuracy						100%	96.67%

BPNN for BRPH amount classification	1	22	0.05	0.8	25,000	52.8%	44%
	2	25	0.05	0.8	25,000	42.4%	52%
	3	25	0.05	0.8	25,000	48.8%	52%
	4	24	0.05	0.8	25,000	45.6%	44%
	5	25	0.05	0.8	25,000	47.2%	48%
	6	24	0.05	0.8	25,000	56.8%	44%

Average accuracy						48.93%	47.33%

Note: Nodes, the number of nodes in the hidden layer; LR, learning rate; DF, dynamic factor; MTT, the maximum number of training times.

**Table 6.** Response features of the sensor array.

**Number in Array**	**Sensor Name**	**Object Substances for Sensing**	**Threshold Value (mL·m**^−^**^3^)**
R1	W1C	Aromatics	10
R2	W5S	Nitrogen oxides	1
R3	W3C	Ammonia and aromatic molecules	10
R4	W6S	Hydrogen	100
R5	W5C	Methane, propane and aliphatic non-polar molecules	1
R6	W1S	Broad methane	100
R7	W1W	Sulfur-containing organics	1
R8	W2S	Broad alcohols	100
R9	W2W	Aromatics, sulfur- and chlorine-containing organics	1
R10	W3S	Methane and aliphatic	10
